# Reply to Witthöft et al. Comment on “Wardzinski et al. Mobile Phone Radiation Deflects Brain Energy Homeostasis and Prompts Human Food Ingestion. *Nutrients* 2022, *14*, 339”

**DOI:** 10.3390/nu14142950

**Published:** 2022-07-19

**Authors:** Ewelina K. Wardzinski, Kamila Jauch-Chara, Sarah Haars, Uwe H. Melchert, Harald G. Scholand-Engler, Kerstin M. Oltmanns

**Affiliations:** Section of Psychoneurobiology, Center of Brain, Behavior and Metabolism, University of Luebeck, Ratzeburger Allee 160, 23538 Luebeck, Germany; kamila.jauch-chara@uksh.de (K.J.-C.); sarah.haars@medizin.uni-leipzig.de (S.H.); uwe.melchert@uni-luebeck.de (U.H.M.); kerstin.oltmanns@uni-luebeck.de (K.M.O.)

We are somewhat surprised about the extent of the feedback that we received upon our publication [1], in terms of the not entirely new connection between mobile phone radiation, brain activity, and food intake, being previously explored by EEG, association studies, and animal experiments (as outlined in the introduction of our paper). Ten years ago, scientists found “alarming” evidence of a long-term association between mobile phone radiation and obesity in humans [2]. Specifically, we are perplexed by the partly emotional character of the discussion among our readers. However, back to the facts:

We thank our scientific colleagues for their detailed analyses and considerations [3] regarding our study and are pleased to explain the open points for more clarity.

First of all, we would like to address the annotated sample size. We agree with the authors that prior to a generalization of our findings, further experiments, e.g., in women, need to be conducted. Notwithstanding, it is a widespread misconception that a participant number of *n* = 15 would generally not be sufficient for scientific evidence in studies. Indeed, this would certainly be the case if we had compared several different groups with each other and thus the interindividual variance was correspondingly large. However, our study was performed as a crossover design, i.e., each subject underwent all three conditions and was thus his or her own control. In fact, with a crossover design, a considerably smaller subject group is required to meet the same level of statistical power (in terms of type I and type II risk of error) as compared to group comparisons. This is because typical comparability problems between several groups of subjects, which are intrinsically tied to an inherently huge interindividual variance, are precluded in a repeated measures design. On the contrary, the presence of high statistical significance in a smaller participant group is per se evidence of a sufficiently high power and thereby a sufficient number of subjects in the sample. Furthermore, it is statistically well-known that a large effect also becomes significant more likely. An increase in the sample size is therefore neither necessary, nor scientifically justifiable, nor ethically permitted (see the current scientific discussion regarding overpowered studies). Moreover, we have a wide experience in divergent measurements of food intake and cerebral energy metabolism in human experimental studies, which underlies the determination of a required sample size for sufficient power [4,5,6,7,8,9,10,11,12,13,14].

Next, Witthöft et al. critically suggested that one control condition in relation to two exposure conditions would increase the probability of undergoing a verum condition on the first experimental day. They hypothesize that our study participants were consistently more hungry and thereby consumed more calories on the first day irrespective of the condition. Apparently, the authors argue that a study should have several control conditions. However, we consider this rather unusual and have never heard of such a multiple-armed study design, in which as many sham conditions as interventions are conducted (e.g., in the case of a four-armed study design, needing four control conditions). In principle, it is not plausible to us why participants should have eaten more on the first experimental day at all. Apart from the fact that this is speculation, two main points argue against the assumption that the increased food intake is associated with an order effect. First of all, the suspected effect of order would not explain why 13 of 15 participants consumed equally more calories in both exposition conditions, i.e., they consumed more food on two days of the study, not only on the first day as Witthöft et al. believe. Moreover, our participants primarily consumed more carbohydrates and displayed cerebral metabolic alterations in both stimulation conditions, which also cannot be explained by a first-day effect.

The third point raised by Witthöft et al. was a criticism of the blinding. Indeed, we had discussed the single blinding when creating the study design. In fact, there were some arguments that convinced us that a complex double blinding would not be necessary: First of all, subjects were completely unaware of the aim of the study, i.e., measurements of their food intake as a primary outcome measure (please see the methods section). Given that our participants had, however, realized that the buffet testing was the most important part of the study, they must unanimously have decided to consume more calories in both radiation conditions (and even equally more upon both mobile phone expositions). Moreover, they must have agreed upon eating significantly more carbohydrates in the verum conditions than after the sham. We think that this is a rather unlikely scenario. Another point is a deliberate increase in the measured high-energy phosphate content in both radiation conditions, as Witthöft et al. apparently assume. We cannot believe that this would be possible even if our single blinding had not worked out in our study. Another argument in this context is a previous work by Henz et al. (also from Mainz University) [15], who demonstrated in a double-blind study that human brain activity as induced by mobile-phone-emitted electromagnetic fields is significantly increased, which is in accordance with our findings on brain metabolism. Our final conclusion is that so many improbable assumptions are linked with each other in these considerations that a bias due to single blinding can be ruled out.

The final point put forward by Witthöft and colleagues relates to the assumption that EMF effects are locally circumscribed and that exposure of the right temporal region does not affect other regions of the cortex. In fact, there is evidence from a previous study that unilateral alterations in high-energy phosphates can be identically observed on the contralateral hemisphere of the brain, which argues for a more widespread effect in other regions than just a circumscribed part of the brain [4]. However, Witthöft et al. are right that we did not perform any measurements within the hypothalamus which could prove that this region was affected by EMFs. However, on the other hand, one cannot exclude that this was the case as the authors assume. Since previous studies have shown a relationship between brain energy metabolism and body weight [14], as well as food intake [6,11], it is reasonable to hypothesize that the increased calorie intake is due to cerebral high-energy phosphate alterations induced by the mobile phone radiation in our study. However, EMF exposure does not necessarily need to directly stimulate the deep hypothalamic region; it is also conceivable that EMFs initiate an activation of the brain via a corresponding increase in general energy expenditure, which is sensed within the hypothalamic area and subsequently compensated for by an increased food intake. However, our study was a typical proof-of-principle inquiry. We demonstrated significant effects of EMFs on food intake behavior and cerebral energy metabolism. The underlying mechanisms cannot be unraveled by our human experimental approach. This will be a challenge for future studies.
